# Supplementary material for: Identifying Factors to Facilitate the Implementation of Decision-Making Tools to Promote Self-Management of Chronic Diseases into Routine Healthcare Practice: A Qualitative Study
Source: Healthcare (Basel). 2023 Aug 25;11(17):2397. doi: 10.3390/healthcare11172397 (PMC10487156; doi:10.3390/healthcare11172397)
Supplement: Supplementary file 1 [file healthcare-11-02397-s001.zip › healthcare-2508891-supplementary.pdf]

Supplementary Materials Document S1– Semi-structured interview guideline

Number	1. Question for decision-makers to interrogate concept<br>1.1 Questions to identify tangible enabling factors
1	What are the different roles and responsibilities within your team for decision tool use?
1.1	Who should be involved in the clinical process to use the decision tools?
2	Can you talk about the role of administrative staff in supporting decision tool use?
2.1	How do you build enthusiasm and support among administrative staff for a new process or way of working?
3	How do you ensure that the whole team understands the purpose of a new process?
3.1	How do you develop a shared understanding among your team about a new way of working?
4	Tell me about the role of clinical leaders in implementing new processes or ways of working, like decision tools
4.1	What makes healthcare professionals want to drive changes in ways of working?
5	Does the use of decision tools align well with your organisational priorities?
5.1	How should decision tool use be measured?
6	How far do the use of decision tools align with the way of working in your country's health system?
7	Does your reimbursement model affect the likelihood of decision tool use?
7.1	Is the use of decision tools encouraged or discouraged by the way your work is funded?
7.2	What non-financial incentives would you need (legal requirements, patient welfare (purpose), workflow facilitation)? laws, policies, compensation)?
8	Does your reimbursement model (capitated, or FFS) capture activities like using a decision tool?
9	Does your country or region's health system performance assessment model take account of decision tools?
10	Does the use of a decision tool by your provider have implications for other providers in your local system?

Number	1. Question for healthcare professionals to interrogate concept<br>1.1 Questions to identify tangible enabling factors
1	What type of patient is suitable for the use of a decision aid?
1.1	At what stage of a disease are such decision aids relevant for patients?
2	What are the different roles and responsibilities within your team for decision tool use?
2.1	Who is important for the decision tool use in practice?
3	Can you talk about the role of administrative staff in supporting decision tool use?
3.1	How do you build enthusiasm and support among administrative staff for a new process or way of working?
4.	Tell me about the role of clinical leaders in implementing new processes or ways of working, like decision tools
4.1	What makes healthcare professionals want to drive changes in ways of working?
5	How do you ensure that the whole team understands the purpose of a new process?
5.1	How (what activities and strategies) do you develop a shared understanding among your team about a new way of working?
6	How comfortable are you with using a decision tool?
6.1	What training or support is needed to build confidence in using decision tools?
7	How important is it that patients have prepared themselves to use the PtDA in a clinical consultation?
7.1	Tell me how to prepare patients to use a new tool like a PtDA
8	How should patients be invited to use the PtDA while in a consultation?
9	At what point do healthcare professionals need to be informed and engaged about a decision tool?
9.1	When and how should healthcare professionals first see the decision tool?
10	Do you see yourself or your team as the most appropriate setting to use a decision tool, compared to another team or provider?
10.1	Who is the most appropriate healthcare professional to use a decision tool?
